# On the Injuries to Health Occasioned by Breathing Impure Air in Close Apartments

**Published:** 1842-04

**Authors:** 


*On the Injuries to Health occasioned by breathing Impure Air in close apartments.*—The *Lancet* for June 19th, 1841, contains some remarks on this subject by Dr. Elmore, which we conceive to be of so much importance, that we shall transfer the greater part of them to our pages, and invite to them special attention. The entire neglect, for the most part, of all means for ventilation in our buildings, public as well as private, and the imperfect means adopted for the purpose in the few instances in which it has attracted some little consideration, is truly remarkable when it is considered that a constant and duly regulated supply of atmospheric air is essential to the sustention and prolongation of life, and to mental enjoyment. Formerly our dwellings in Philadelphia were warmed (if so it might be called) by wood fires, the greater part of the heat from which was carried up the huge chimneys, and the currents of air thus necessarily created, were supplied by the crevices in the doors and windows. The consequence was, a general and just complaint by those who collected in front of the fire that their backs and feet were freezing whilst their faces were burning.

At present warming houses by means of heated air is becoming prevalent, and whilst ingenuity has been taxed for the means of effecting this with most economy, i. e. of heating the air to the highest temperature, the *quality* of this air and the means by which it is to pass off when rendered more impure than when received, seems not to have obtained a thought. The air is heated by *iron* stoves in chambers of masonry. These chambers are too small, and the air is conveyed through the building by flues also too small. Hence it is necessary in cold weather that the stove should be kept red hot in order to heat the air sufficiently to warm the house, and thus the air is rendered almost unfit for respiration when introduced in the rooms. No means being provided by which this air shall regularly pass off, it becomes confined, and much discomfort and many ill effects result from breathing this air, constantly becoming more and more impure. But Dr. Elmore has discussed this so fully; that we shall not dilate further on the subject, but content ourselves with quoting his observations.

A free admission of air, and a constant supply and free circulation of this element, "is as necessary for sustaining life as a given quantity for the combustion of the fuel we require to warm our apartments: our builders, nevertheless, only provide for the latter, as if the former, although the more important, was of minor consideration; or, that they conceived the chimney-draught sufficient for both purposes, when in reality it does not answer that for which it is principally intended; as by far the greater portion of the heat generated in our open fire-places is carried up the chimney, by sharp currents of air from occasional openings of doors, or such crevices as it may force its way through; being, moreover, frequently productive of serious bodily injuries, particularly to those of delicate frames; while it cannot be sufficient for the purposes of wholesome ventilation: this air being colder than that already in the room, is consequently of greater specific gravity, and must form a lower stratum, not unfrequently felt by those placed round the fire, suffering from an undue proportion of heat at one side and of cold at the other.

"It should also be borne in mind that the openings of our fire-places being seldom more than three or four feet from the floor, the upper stratum of air which we breathe is neither removed nor purified by this under-current, and must, from being breathed over and over again, be productive of most prejudicial effects, and that the contamination of this atmosphere is considerably augmented at night by the combustion of lights. It has been ascertained that the quantity of air breathed by an ordinary-sized person is about two thousand cubic feet per hour; and that two mould candles consume as much of the oxygen of this air as a human being; and that the nitrogen and carbonic acid gas which remain are peculiarly inimical to animal life, and that when carried up by the currents occasioned by combustion and respiration, they form an upper stratum where they remain, and must be repeatedly inspired before they make their escape into the chimney, the only ventilating flue with which our houses are provided.

"It should also be observed, that the heat thus generated is in proportion to the quantity of oxygen abstracted from the atmosphere, which enters into combination with the carburetted hydrogen of the flame of candles, coal-gas, oil, or other inflammable matter, from which light is produced. That every cubic foot of carburetted hydrogen consumed, unites, on an average, with two cubic feet of oxygen, (that portion of the atmosphere required to support animal life,) and that the product of this combustion is about two and a half inches of water and one of carbonic acid gas, which, when inhaled in its pure state, proves instantly fatal; and the greater the proportion we inhale, in addition to the vapors evolved from the lungs and skin, the more pernicious the effect.

"Supposing, for example, that the perfect lighting of an ordinary-sized apartment requires fifteen cubic feet of carburetted hydrogen per hour, this would form about a pint and a half of water, and fifteen cubic feet of carbonic acid gas; for whenever carburetted hydrogen gas is burned with oxygen, or atmospheric air, these are the products of the combustion, whether the carburetted hydrogen is obtained from wax, tallow, oil, or coal. If, therefore, this lighting continues in an unventilated apartment for seven hours, one gallon of water is produced, the greater part of which will be deposited on the walls, windows, furniture, polished metal, or other cold surfaces, with which it comes in contact: and to some articles of this nature it is known to prove highly prejudicial, in addition to the in jury to health occasioned by an increased quantity of moisture, mixed with the air we breathe. As one of the principal functions performed by this air for the preservation of health, is to carry off with it a considerable quantity of vapor, in order to prevent its undue accumulation in the lungs, it is, therefore, evident, that after it has been already so loaded it cannot properly perform these functions, and that consumption and other complaints are thus frequently induced.

"The prejudicial effects of carbonic acid gas (which is the same as the choke-damp of mines) as well as the nitrogen of the air, which is set free by the abstraction of the oxygen (and amounts in quantity to four times that of the oxygen), are well known, and ought by all possible means to be provided against. This has been attended to within the last few years in our public hospitals, and the mortality in consequence considerably decreased; and likewise in several of our manufactories and public establishments, where the diseases generated by the number of persons congregated in such establishments have been proportionably diminished. In the House of Commons, also, where hundreds of members, with hundreds of candles burning at night, tended so much to vitiate the atmosphere, important improvements in lighting, as well as ventilation, have been recently made; but in our domestic establishments little or no attention has been paid to this important subject, and the foundation of a variety of diseases must be the result, particularly from the foul air breathed at balls, or other crowded assemblies.

"The confinement of air in our churches and places of public worship must also be highly prejudicial, as we are frequently exposed to an atmosphere, on entering one of these edifices in the summer months, ten or fifteen degrees below that of the external air, independent of the stagnant state in which it has been allowed to remain during a whole week, often vitiated, in a greater degree, by the gaseous matter evolved from human remains; and even in private houses much inconvenience is experienced from the stagnant state of the atmosphere in close and gloomy weather, as the entire basis of ventilation depends on the possibility of producing a constant circulation as well as supply of this element. Close stoves are also objectionable, when made of iron, and heated to a certain temperature, as oxide of iron is produced by the powerful attraction of that metal for oxygen, and the formation of ammoniacal gas by the mixture of the nitrogen which remains, with hydrogen, acting on our bodies and olfactory nerves.

"But if stoves were constructed of masonry throughout, as in many other countries, or of fire-tiles, or porcelain plates, imbedded in mortar, with well-regulated flues, they would be far preferable to open fire-places; this substitution of imperfect conductors of heat being not only consistent with the soundest principles of economy in the preservation of heat, and its more uniform distribution through apartments, but more conducive to health than bringing the air in contact with iron stoves or pipes. Our desire, however, for polished metals in almost every department of our domestic appendages, united to the interests of the furnishing ironmongers, to whom these matters are usually left, must operate, in no small degree, in determining the prevailing taste for this commodity. Porcelain stoves may, nevertheless, be made sufficiently ornamental for those who prefer health to fashion; and when apartments are provided with well-regulated apertures and flues through their ceilings into the adjoining chimneys, to carry off the air vitiated by respiration and combustion, a sufficient degree of heat may be obtained with a sufficient supply of that element, without which it is impossible to maintain health.

"The healthy appearance of those who pass the greater part of their time in the open air, sufficiently indicate its advantages. Armies are also well known to have greater numbers on the sick list when well-housed, and what is considered comfortably settled in quarters, than when exposed in a campaign to the vicissitudes of the seasons for weeks and months, without any other covering than the canopy of heaven, or occasionally of a tent or hut, or the shade of a tree. These facts ought to satisfy us that we should admit the air as freely as possible, and provide, at the same time, for its escape through the ceilings of our apartments at all seasons of the year, as the temporary and often imaginary inconvenience of a little cold, when compared with the decided disadvantages of breathing impure air, is by far the lesser evil."—*Am. Jour. Med. Sci.*

